# Evaluation of Hand–Dug Wells in Rural Haiti

**DOI:** 10.3390/ijerph15091891

**Published:** 2018-08-31

**Authors:** Hayley E. Schram, Peter J. Wampler

**Affiliations:** Department of Geology, Grand Valley State University, 1 Campus Drive, Allendale, MI 49401, USA; schramh@mail.gvsu.edu

**Keywords:** groundwater, Haiti, filtration, water treatment, developing country, point of use treatment, household treatment

## Abstract

Water resources, especially safe, potable water, are limited for many Haitians. In areas where shallow groundwater is available, many household water needs such as laundry, bathing, and cooking are supplied by hand–dug wells. In order to better understand the water quality and prevalence of these household wells, 35 hand–dug wells were surveyed and sampled near the Hôpital Albert Schweitzer in Deschapelles, Haiti. Water samples were collected and tested for fecal coliform and *Escherichia coli* using the IDEXX Colilert–18 method. Of the samples collected, 89 percent were determined unsafe to use as a drinking water source based on the World Health Organization standard of 1.0 colony–forming unit (cfu) *E. coli* per 100 mL. Sixty–six percent of the wells exceeded recreational/body contact standards for the state of Michigan (130 cfu/100 mL). Some of these wells were deemed suitable for conversion to a new well type called in situ filtration (ISF) wells. In situ filtration wells are installed with an internal sand filter pack, PVC casing, pump, and cap which seals the well from surface contamination and provides additional water treatment as water is pumped. Previous ISF installations have reduced *E. coli* to safe drinking water levels within 90 days.

## 1. Introduction

According to the World Health Organization, 663 million people worldwide use unimproved water sources, including springs, surface water, and unprotected wells [1]. Many developing countries like Haiti lack water access due to limited water resources, infrastructure, contamination, and/or political instability. Haiti is located on the western one–third of the island of Hispaniola, has a population of roughly 10.7 million people. It is divided into three regions: the northern, central, and southern regions. The central region largely consists of the Artibonite Valley, named after the Artibonite River, which is the longest river on the island. This river, which serves as a collector for contamination through tributary rivers and streams, contains high concentrations of bacteria including coliform, *Escherichia coli* (*E. coli*), and *Vibrio cholorae* (*V. cholerae*) [2]. Hand–dug wells located near the river and on the floodplain act as primary water sources for many Haitians living near the Artibonite River. In rural mountain areas, women and children walk, often multiple times a day, to retrieve water in buckets, plastic bottles, or jugs from springs. Water quality in these springs is also poor due to elevated *E. coli* levels [3]. Hand–dug wells within the study area are primarily Hand–dug wells are typically used for water needs such as cooking, laundry, and bathing.

**Figure 1:**
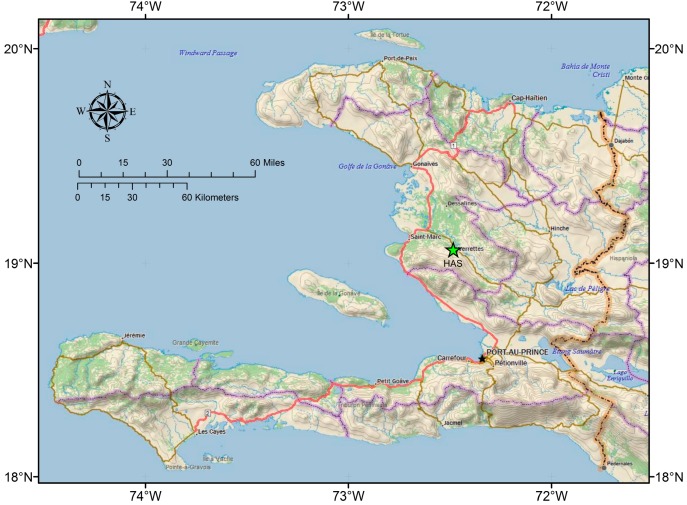
Haiti location map [3].

**Figure 2:**
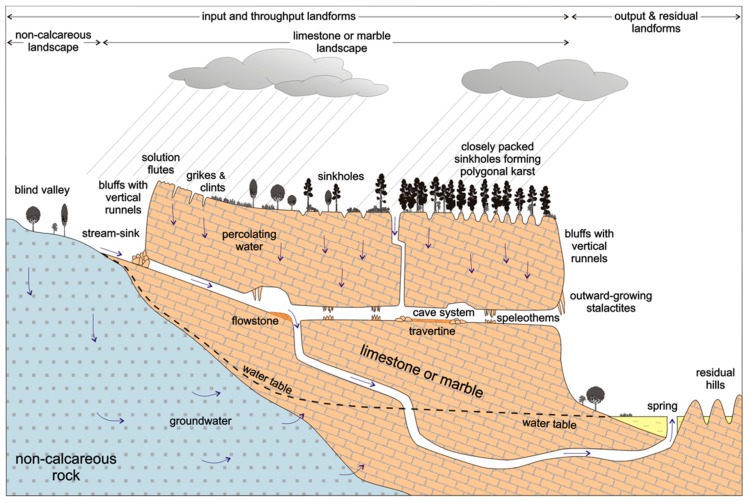
Karst topography dissolution pathways [7].

In the Artibonite Valley region, near Verrettes (Figure 1), the implementation of safe water interventions is complicated by cultural and social practices that make some types of interventions less effective [4,5,6]. The dissolution of bedrock results in the creation of pathways where unsafe and contaminated water in shallow aquifers can spread and further contaminate springs, caves, and other karst aquifers (Figure 2) [3,7]. In 2010, Haiti was the most underserved country in the western hemisphere in terms of water and sanitation infrastructure [8]. Despite this, sustainable effective treatment methods remain problematic. Point–of–use (POU) water treatment methods have become the preferred method in many developing nations to improve water quality by treating water in small batches in the home [9]. Methods such as Sawyer filters, biosand filters (BSFs), chlorination tablets, and boiling water are the most popular practices among Haitians. Sawyer fiber–membrane filter technology is promising for in–home treatment due to its low cost and potential longevity [10].

In 2005, a field study of 107 households was conducted to evaluate the performance of Manz Concrete BSF. In a five–year period, nearly 2000 BSFs were installed around Deschapelles by Hôpital Albert Schweitzer (HAS). Through networking, conducting interviews, and making observations, it was found that the average lifespan of a BSF was 2.5 years. By conducting water sampling, it was found that 97 percent of the BSF samples contained 0–10 *Escherichia. coli* cfu per 100 mL. The hand–dug wells located near these households contained an average of 234 *E. coli* cfu per 100 mL [11]. In a follow–up study conducted in 2011, 55 BSF were visited and assessed. These BSF were previously installed by three separate organizations. Of the 55 BSF installed, only 29 (53 percent) remained in use at the time of the sampling [4]. Eighty–six percent of the water treated by the BSF contained *E. coli* concentrations of less than 10 most probable number (MPN) per 100 mL, suggesting the filters were still effective when in use. In addition to BSF methods, other water treatment interventions have been implemented to provide safe and sustainable water including solar water disinfection (SODIS), reverse osmosis, ultraviolet disinfection, ozone disinfection, Sawyer filters, and a new treatment method described here as an in situ filtration (ISF) well. The ISF wells are a passive means of water treatment that may provide an extra layer of protection for homes that either do not have in–home treatment, or for whatever reason, are choosing not to utilize any treatment method to provide safe drinking water.

**Figure 3:**
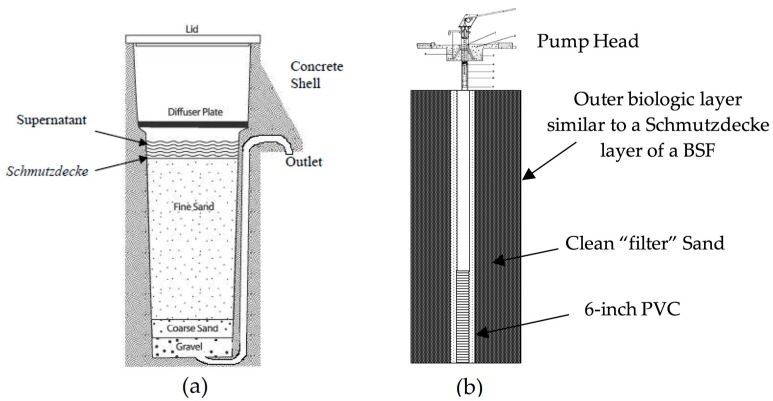
(**a**) Biosand filter (BSF) components [12]; (**b**) in situ filtration (ISF) well components.

An ISF well uses a sand pack to filter water slowly before it is drawn through the well screen. In situ filtration wells may enhance natural biological activity within the outer layer of the well similar to the Schmutzdecke layer in a BSF [12]. Since most of the bedrock in this region is limestone, over time rock is dissolved, creating pockets where pathogen populations can dwell. This results in contaminated groundwater and aquifers. Better water quality is often obtained by installing deep wells into the underlying aquifer as a means of bypassing the surface contamination, and improved sanitation methods to reduce surface contamination. An ISF well costs roughly one–tenth the price of a traditional drilled well, and the materials can be found locally in country. The ISF wells are similar to those described by others as “bank filtration wells” [13,14]. In situ filtration wells are a relatively new application of “bank filtration” that uses a combination of polyvinyl chloride (PVC) casing and a natural sand pack to reduce *E. coli* levels (Figure 3).

The goals of this study were to (1) collect bacterial contamination data on hand–dug wells; (2) determine common practices being used to cover or protect these wells; (3) evaluate potential contamination sources; (4) measure the amount of water volume in hand–dug wells; and (5) discuss possible treatment methods for water in these wells to make is safer for residents. Studying hand–dug wells provides residents with more complete information regarding hand–dug well water quality and treatment methods that are sustainable and economical.

## 2. Materials and Methods

**Figure 4:**
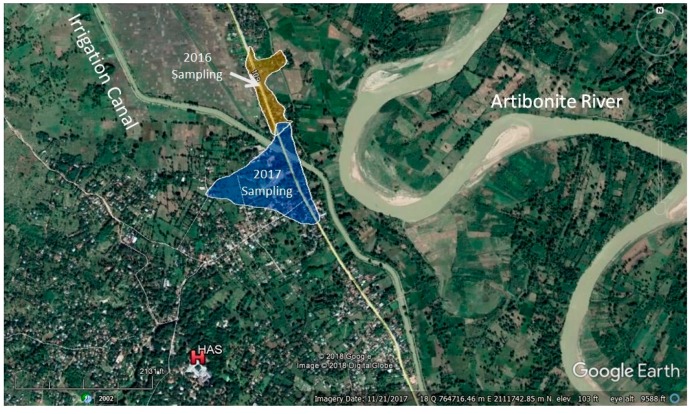
Hand–dug well sampling areas in 2016 and 2017.

Data was collected using pseudo–randomized sampling of 35 hand–dug wells (20 wells in June 2016 and 15 wells in May and June 2017) located in Deschapelles and Borel, Haiti (Figure 4). This process involved locating a hand–dug well, asking the homeowner’s permission to sample the well, recording well information (including the owner’s name and the amount of water seasonally withdrawn from the well), and concluded by inquiring about additional locations of hand–dug wells in the area. Deschapelles and Borel were selected due to the high density of hand–dug wells and close proximity to HAS where the bacterial testing would take place.

### 2.1. Physical Characteristics

The distance between the hand–dug wells ranged from 0.1 to 0.5 kilometers (km) in 2016 and 2017. Global positioning system (GPS) locations were determined at each well using a Garmin GPSMap 64s GPS (WGS84). Each well was photographed using a Nikon Coolpix AW130 and georeferenced. A physical description was recorded for each well consisting of the type of well collar, and general surroundings of the well. A Bosch laser distance meter (DLE40) in addition to materials improvised in the field including sticks and rope were used as measuring devices. Measurements included total well depth, static water depth, and depth from the ground surface to the water surface. All well measurements and water samples were obtained during the rainy season. Water depth measurement were made to calculate volume prior to removing water samples.

### 2.2. Bacterial Testing

**Figure 5:**
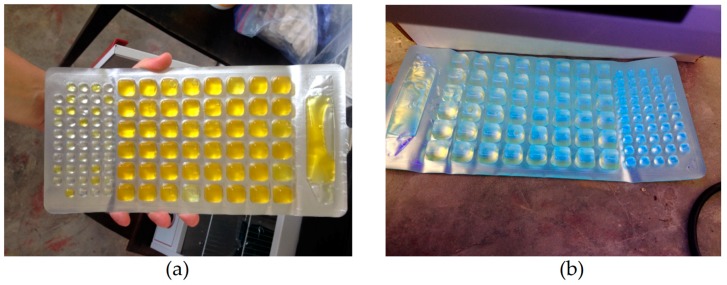
IDEXX Colilert–18 Quanti–Trays: (**a**) water sample testing positive for coliform; (**b**) water sample under UV light, testing positive for *E. coli*.

One hundred mL water samples were taken at each hand–dug well location (*n* = 35). These samples were evaluated for total coliform and *E. coli* contamination using the IDEXX Colilert–18 Quanti–Tray System (IDEXX Laboratories, Westbrook, ME, USA). Water samples were collected using sterile 100 mL Whirl–Pak^®^ bags (NASCO, Atkinson, WI, USA), and were transported back to HAS in Deschapelles for bacterial analysis, within two hours of being sampled. The process began by adding a Colilert–18 reagent to each individual 100 mL Whirl–Pak^®^ sample until it fully dissolved. The mixture was placed in a Quanti–Tray, which was sealed and incubated at 35 ± 0.5 degrees Celsius for 18–24 h. The results were read using the IDEXX results table, where the number of colored and fluorescing large and small cells determined the MPN for *E. coli* and coliform bacteria (Figure 5). Control samples of filtered Culligan^TM^ water were included with well samples to evaluate cross–contamination. Control test results were negative for *E. coli* contamination, and positive for total coliform in one case.

### 2.3. ISF Well Monitoring

Installation of the ISF well sampled was accomplished in 2013 using local labor and materials with the exception of a cast iron pitcher pump which was transported from the United States [15]. Filter sand was obtained from a nearby stream and sifted using hand–made sieves with a roughly 1–2 mm sieve diameter. The ISF well was completed and sealed with locally derived clay prior to concrete finish work to install the pump. Water samples for bacterial analysis were collected in sterile 100 mL Whirl–Pak^TM^ bags at random time intervals from the pump and analyzed using the IDEXX methods described above.

## 3. Results

**Figure 6:**
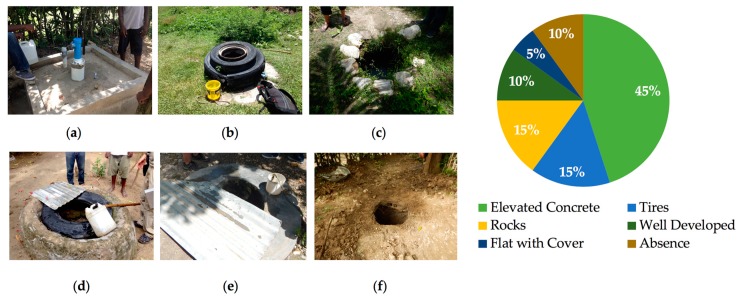
Hand–dug well collar variations: (**a**) elevated concrete; (**b**) tires; (**c**) rocks; (**d**) well developed; (**e**) flat with cover; (**f**) no collar/absence.

Detailed well characteristic data were collected in 2016 for 20 wells. The physical appearance, and the dimensions of each of the 20 hand–dug wells varied minimally from well to well. The well collar, which guards the well from surface runoff contaminants, was the most variable among wells. They included single or multiple tires, medium to large rocks, elevated concrete, well–developed collars with square concrete molds, flat covered wells, and no collar. The 2016 percentages of each collar type from our samples (*n* = 20) are shown in Figure 6. Of the 20 wells sampled, 9 (45%) were found to have an elevated concrete collar, and only 2 (10%) had no collar. From our survey, we found no correlation between collar type and decreased amounts of coliform or *E. coli* contamination.

**Figure 7:**
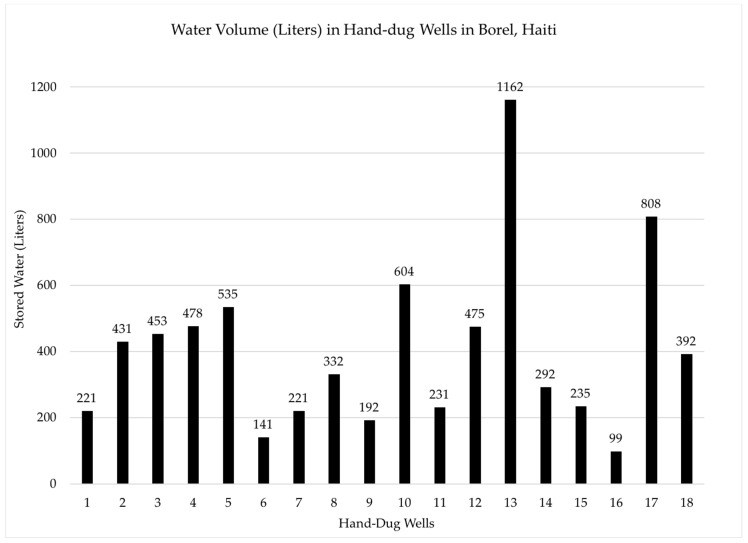
Hand–dug well water volume data.

Total well depth, and depth to water were used to calculate the volume of water present in each well in 2016 (*n* = 20) (Figure 7). The average volume per hand–dug well was 0.406 cubic meters, or 406 L. No data was collected for 3 wells (sample numbers 10, 11, and 17). Of the 35 hand–dug wells sampled in 2016 and 2017, 30 (86%) of wells exceeded the maximum detection limit (>2419.6 MPN/100 mL) for total coliform, and 4 (11%) exceeded the detection limit for *E. coli*. Table 1 summarizes average and geometric mean for coliform and *E. coli* bacterial testing. A geometric mean was included due to the wide range of *E. coli* MPN values (0–2419.6) using the IDEXX method.

The World Health Organization (WHO) Guidelines for Drinking Water Quality recommend that there should be no detectable *E. coli* bacteria (or the fecal indicator bacteria (FIB)) in a 100 mL drinking water sample [16]. However, in many low–income countries, including Haiti, reports have shown presence of fecal contamination in improved sources, such as piped water, which often exceeds the WHO Drinking Water Quality recommendation [17]. Thirty–one of the 35 (89%) hand–dug well samples exceeded the WHO standard for *E. coli* in 100 mL water samples. *Escherichia coli* in this area not only exceed drinking water standards, but many samples exceeded recreational/body contact standards. In the state of Michigan, the Department of Environmental Quality (DEQ) 30–day geometric mean is 130 *E. coli* per 100 mL [18]. Twenty–three of the 35 (66%) samples exceed this value.

## 4. Discussion

Based on the widespread *E. coli* contamination of hand–dug wells, exceeding Drinking Water Quality recommendations, and in many cases body–contact standards, these wells should not be used for drinking, laundry, bathing, or cooking purposes without treatment. Using the water for laundry and cooking are possible if adequate precautions are taken. Precautions would include WaSH adequate use of chlorination for laundry and adequate boiling for cooking uses [19]. Additional education in safe water practices could reduce the risk for coliform and *E. coli* contamination spread through food preparation and laundering practices.

Because hand–dug wells are heavily relied upon by Haitians in this region, especially during the rainy season, it is important to determine the quantity of water available for use. Data collected in 2016 was used to establish an estimate of well water volume available from hand–dug wells for each household. Available water would vary considerably throughout the year as a result of rainy and dry seasons. Based on measurements made in June, 2016 the average amount of water in wells was 0.406 cubic meters (406 L). Assuming approximately 3.4 people per household, this would represent approximately 119 L per person [20]. According to the United Nations Development Program, the daily amount of water that a family of four uses in Haiti for household needs is approximately 20 L (4 L per person), while in the United States a family of 4 uses 2300 L (575 L per person) for daily needs [21]. These data suggest that the volume of water within hand–dug wells during the rainy season is more than adequate to supply the water needs for a family of four, although as noted earlier this water is not suitable for drinking or many other uses without proper treatment.

**Figure 8:**
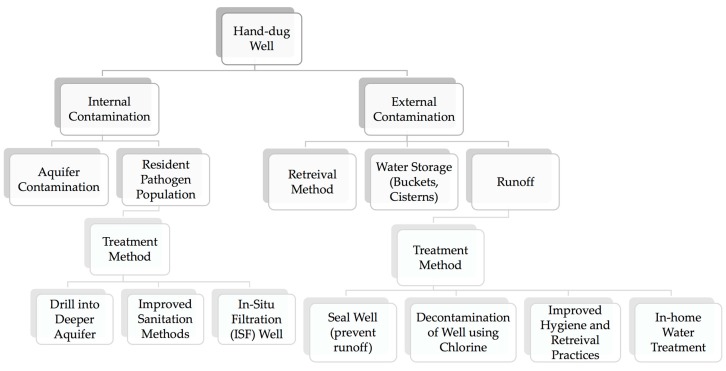
Flow chart depicting contamination pathways and treatment methods.

**Figure 9:**
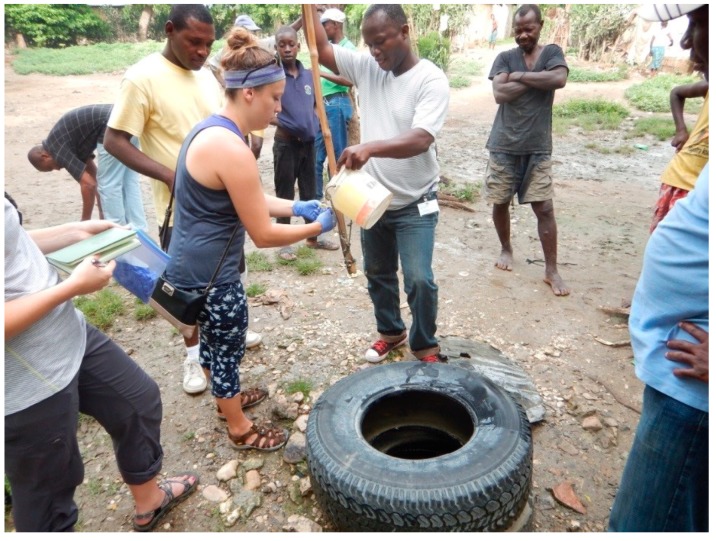
Bucket retrieval methodology.

Observed bacterial contamination of hand–dug wells may be the result of external or internal sources (Figure 8). External contamination occurs when unsuitable water retrieval and storage methods are used that introduce contamination as water is removed from the well. Common retrieval methods in Haiti include the use of plastic buckets attached to ropes which are lowered down to collect water (Figure 9). Similarly, long–term water storage basins including cisterns, buckets, and tanks can introduce and harbor pathogens. Many people have implemented the use of well collars made from rocks, tires, and concrete which provides a modest barrier for contamination from surface runoff entering the well. Internal contamination is another possible source of observed bacterial contamination. Bacteria introduced to the groundwater through poor sanitation can travel to wells through the aquifer from other wells or from more distal sources. It is also possible that warm groundwater (average of 26.5 °C measured in water from springs) and porous aquifers can result in resident pathogen populations [3].

**Figure 10:**
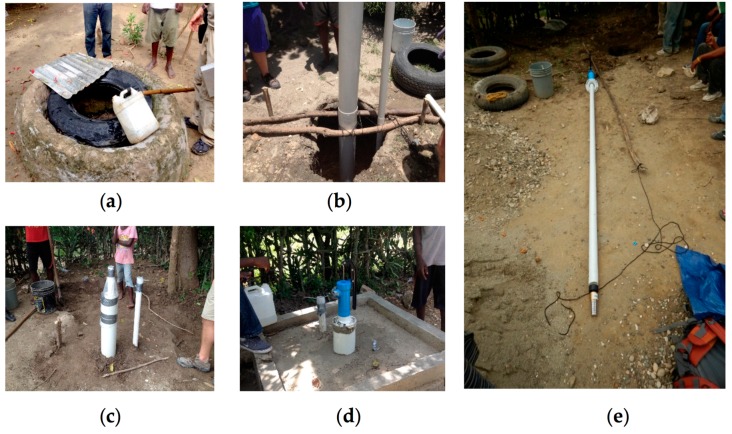
ISF well conversion process materials: (**a**) initial hand–dug well; (**b**) installation of PVC casing; (**c**) sand pack addition; (**d**) final ISF well product with a concrete collar; (**e**) internal ISF well components including a pump head, PVC casing, and check valve.

One possible way for residents of Haiti to treat water removed from hand–dug wells is through the conversion of open wells to sealed ISF wells. In situ filtration well construction utilizes materials that are found in–country and can be constructed with local labor and expertise promoting economic development in rural Haiti. Using PVC casing, a natural sand pack, and basic monitoring well components (i.e., PVC screen, well caps, valves, and a hand pump) hand–dug wells can be converted into an ISF well which will produce treated water that meets WHO drinking water standards in 40 days (Figure 10).

**Figure 11:**
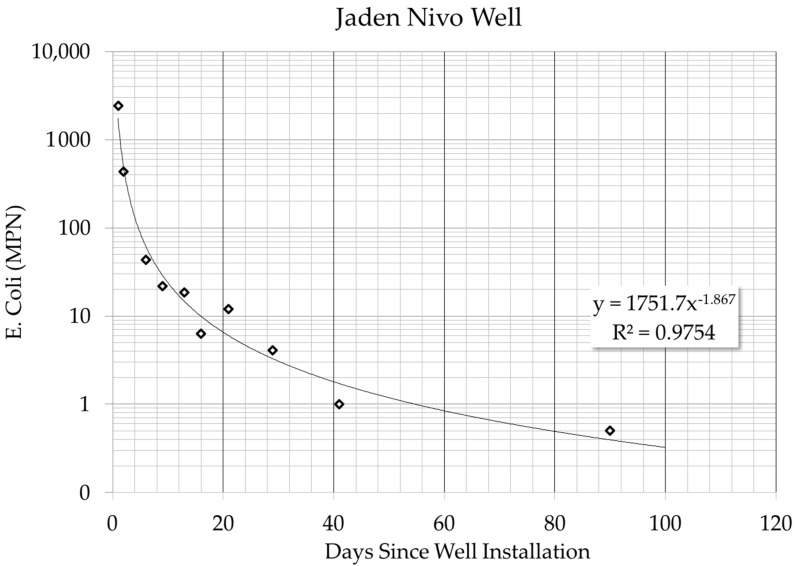
Jaden Nivo ISF well data.

In the summer of 2013 a hand–dug well was installed near the Jaden Nivo spring and monitored for over 90 days. Within that time the levels of *E. coli* contamination decreased from >1000 MPN to <1.0 MPN (Figure 11). The Jaden Nivo well shows that ISF well design can be an effective method for reducing bacteria, however, it is not yet clear how the reduction is being achieved. Additional data and ISF well construction under more controlled conditions is needed to evaluate the effectiveness and sustainability of ISF wells. Additional data about the quantity and quality of groundwater supply, recharge, turbidity, and monthly precipitation will help understand how and why ISF wells are achieving bacterial reductions and eliminate potential for failure of future wells converted to ISF wells.

In addition to the implementation of ISF wells, Sawyer filters could provide an effective treatment method for bacterial contamination in hand–dug wells. Sawyer filters and other hollow membrane fiber filters are inexpensive, small in size, easy to use, and require minimal maintenance.

Overall, residents would benefit from more complete information regarding the hand–dug well water quality and treatment methods that are sustainable and economical. In order for any water treatment method to be effective and sustainable it will need to balance appropriate technology, cultural compatibility, and cost.

### Future Studies

In order to better understand how effective and sustainable ISF wells are additional field and lab studies are needed. Additional groundwater flow and bacterial contamination data are needed in the vicinity of the hand–dug well(s). This could include collecting water depths in wells over time using piezometers to determine groundwater flow direction and recharge rates of the aquifers. Another key component of the ISF well is the sand used for filtration. Our study conducted in June 2016 used sand from the Artibonite River, which was not decontaminated prior to installation in the wells. Additional experiments are needed to determine if sand sterilization prior to well installation is needed. This could limit the amount of external contamination introduced to the well during construction.

In order to evaluate the contribution of external contamination on water quality in hand–dug wells that are not converted to an ISF well, a well could be isolated from surface contamination through sealing and installation of a pump without the sand filtration. This would help evaluate the contribution of the sand filtration to the observed ISF effectiveness. Bacteriological data could be collected before and after isolation and compared to nearby wells which either included or excluded the use of a sand filter or other variables. Additionally, wells with and without components decontaminated with bleach could help evaluate the contribution of internal contamination sources.

## 5. Conclusions

The data collected on physical characteristics and bacterial analysis of the 35 hand–dug wells determined that water from these wells, without some form of treatment, is unsafe and unsuitable for most uses including drinking, laundry, bathing, and cooking. Some of these hand–dug wells might be suitable for conversion to a new well type called an in situ filtration (ISF) well. All 35 wells are adequate in terms of water volume for supplying a family of four (roughly 60 L/day) for daily water needs during the rainy season. Surface protection such as concrete collars did not correlate with lower *E. coli* levels.

In order to gain further understanding of hand–dug wells, water quality and quantity additional monitoring and surveying over time are needed. Recharge rates, groundwater flow direction, turbidity, and precipitation data are needed to increase our understanding of how these wells function and become contaminated. This data could be used to determine which wells are compatible for conversion to an ISF well. A larger sample size of wells would help evaluate correlations between water volume, well collars, and seasonal recharge. If Haitians are to continue to use hand–dug wells as a water source, additional treatment, protection, and study is needed. In the meantime, point–of–use treatment methods such as Sawyer filters could provide an effective and sustainable water treatment method, as bacterial contamination was proven to decrease after use.

## Figures and Tables

**Table 1:** Hand–dug well bacterial water quality data.

Sample Type	N =	Average Coliform	Geometric Mean * Coliform	Average *E. coli*	Geometric Mean * *E. coli*
2016 Hand–dug Wells	20	2156.4	1174.3	817.8	218.8
2017 Hand–dug Wells	15	2084.9	1489.7	283.6	25.5
Combined 2016 and 2017	35	2120.6	1322.7	550.7	74.7

* Values of zero were entered as 0.1 for calculation of Geometric Mean.
